# Requirements for a Bespoke Intensive Care Unit Dashboard in Response to the COVID-19 Pandemic: Semistructured Interview Study

**DOI:** 10.2196/30523

**Published:** 2022-04-13

**Authors:** Brittany Davidson, Katiuska Mara Ferrer Portillo, Marceli Wac, Chris McWilliams, Christopher Bourdeaux, Ian Craddock

**Affiliations:** 1 Department of Electrical & Electronic Engineering University of Bristol Bristol United Kingdom; 2 School of Management University of Bath Bath United Kingdom; 3 French Department School of Modern Languages University of Bristol Bristol United Kingdom; 4 University Hospitals Bristol and Weston National Health Service Foundation Trust Bristol United Kingdom

**Keywords:** intensive care, critical care, COVID-19, human-centered design, dashboard, eHealth, disease monitoring, monitoring, ICU, design, development, interview

## Abstract

**Background:**

Intensive care units (ICUs) around the world are in high demand due to patients with COVID-19 requiring hospitalization. As researchers at the University of Bristol, we were approached to develop a bespoke data visualization dashboard to assist two local ICUs during the pandemic that will centralize disparate data sources in the ICU to help reduce the cognitive load on busy ICU staff in the ever-evolving pandemic.

**Objective:**

The aim of this study was to conduct interviews with ICU staff in University Hospitals Bristol and Weston National Health Service Foundation Trust to elicit requirements for a bespoke dashboard to monitor the high volume of patients, particularly during the COVID-19 pandemic.

**Methods:**

We conducted six semistructured interviews with clinical staff to obtain an overview of their requirements for the dashboard and to ensure its ultimate suitability for end users. Interview questions aimed to understand the job roles undertaken in the ICU, potential uses of the dashboard, specific issues associated with managing COVID-19 patients, key data of interest, and any concerns about the introduction of a dashboard into the ICU.

**Results:**

From our interviews, we found the following design requirements: (1) *a flexible dashboard*, where the functionality can be updated quickly and effectively to respond to emerging information about the management of this new disease; (2) *a mobile dashboard*, which allows staff to move around on wards with a dashboard, thus potentially replacing paper forms to enable detailed and consistent data entry; (3) *a customizable and intuitive dashboard*, where individual users would be able to customize the appearance of the dashboard to suit their role; (4) *real-time data and trend analysis* via informative data visualizations that help busy ICU staff to understand a patient’s clinical trajectory; and (5) *the ability to manage tasks and staff,* tracking both staff and patient movements, handovers, and task monitoring to ensure the highest quality of care.

**Conclusions:**

The findings of this study confirm that digital solutions for ICU use would potentially reduce the cognitive load of ICU staff and reduce clinical errors at a time of notably high demand of intensive health care.

## Introduction

### Background

The intensive care unit (ICU) is a busy working environment where a variety of clinical staff perform different duties at scheduled times of the day, while also having to respond to unexpected, often critical issues with patients. ICUs are typically heavily instrumented, and staff need to be alert to many sources of data from equipment such as ventilators as well as the patients’ vital signs and lab test results. For a complex ICU patient (eg, those with multiple conditions), anywhere between 80 and 200 medical interventions are delivered daily and, prior to the COVID-19 pandemic, a member of ICU staff would typically be responsible for up to 10 patients each day [1]. Nonoptimal decisions and clinical errors in this cognitively demanding environment are known to impact patient outcomes [1-4], and a large body of evidence demonstrates that working in an ICU is highly stressful [5,6].

Much of the relevant data for clinical decision-making is already available to staff in the ICU. However, this information is typically scattered across a number of applications, devices, and pieces of paper within the ward. Hence, ICU staff may inadvertently fail to notice signs of a patient’s deterioration and struggle to effectively communicate patient updates (eg, test results, medication) or patient requirements (eg, changing tubes, sedative drug management), which will contribute to worse patient outcomes [3,7]. Additional problems such as equipment failures [3] further add to the complexity of working in the ICU and the importance of clear communication among ICU staff [3,8].

The COVID-19 pandemic has generated unprecedented challenges around the globe [9,10], with particularly detrimental impacts on health care systems [10,11]. Increased hospitalizations from COVID-19 put an additional strain on ICU resources, specifically beds with mechanical ventilation [11,12]. In the United Kingdom, this shortage has been such a concern that additional intensive care capacity was made available by the construction of 11 temporary “Nightingale” hospitals [13]. As the pandemic grew in early 2020, the two local ICUs in University Hospitals Bristol and Weston National Health Service Foundation Trust reported a critical need for an information technology solution to help their staff manage increased patient caseloads. The outline brief from the units envisaged a dashboard that would pull together disparate data sources in the ICU to help reduce cognitive load on extremely busy clinical staff. A particular concern was that staff-patient ratios, and hence patient safety, would be eroded by a combination of massively increased patient numbers and COVID-19 cases among their trained staff.

### Dashboard for COVID-19 ICUs

The development and use of “dashboards” within health care services are becoming increasingly popular [14]. These are typically interactive, visual tools that help ascertain and monitor trends or the status of key indicators of patients’ health condition [15]. We define a dashboard as “a visual display of the most important information needed to achieve one or more objectives […and] are frequently used to consolidate and arrange these data so the information can be monitored at a glance” [16]. The key here is that a dashboard must bring together a variety of data and information for (ICU) staff to understand how unwell a patient is and administer appropriate care efficiently and often under time pressure, especially during a pandemic with increasing numbers of extremely sick patients entering ICUs.

It has been consistently found that the use of various graphical displays of information helps detect adverse events and increases clinical diagnostic accuracy [17,18]. Other work has also found that additional clinical support systems bringing together patient information with electronic health records (EHRs) are associated with reduced hospital and ICU mortality rates, shortened hospital stays, and reduced costs of hospitalization (in the US context) [19]. Despite this, it is reasonable to suppose that clinical dashboards could have an important role to play in delivering health care in the ICU, although it is to be expected that this picture will be quite complex. For example, the utility of a dashboard may be limited at moments of crisis since using it requires attention and free hands; new insights into a patient’s trajectory might indeed be useful or, alternatively, might introduce additional stress for staff, whereby a specific dashboard may fit the needs of senior staff charged with overseeing the operation of the whole unit, but might be less useful for staff caring for a specific patient [17,20]. However, a recent systematic review and meta-analysis [17] found that dashboards or interactive displays are linked to more accurate, and indeed faster, clinical care decisions in critical care/ICUs. ICU dashboards have been used in Brazil to allocate resources and to obtain near real-time information on suspected and confirmed COVID-19 cases [21]; however, we are not aware of any published study that has interviewed clinical staff and broadly captured the requirements for such a dashboard during the pandemic. Such a study will therefore provide both COVID-19–specific and extreme environment–specific working practices and insights [22]. With new highly transmittable COVID-19 variants emerging and with no guarantee that vaccines will engender an immune response against all future variants, it is hoped that the methodology and the findings in this contribution will be of value to those in a position to develop much-needed new technologies for the clinical frontline.

When building tools and devices, it is important to take a human-centered approach to the design of clinical technologies by including the end-user needs as early as possible [14,23,24]. For reasons of efficiency, patient safety, and job satisfaction, clinical professionals should have a direct role in the design of the tools they will use for their jobs [23-26]. Further, this helps to ensure that critical features in design, particularly in terms of visual displays, are not misunderstood [17]. Therefore, this approach helps to ensure that such tools are developed as a result of clinical “pull,” not technological “push,” and helps to prevent technology resistance or avoidance [24,27]. Hence, our approach was to commence interviews with clinical staff within the two local ICUs to elicit their requirements for a clinical dashboard. Due to the extreme pressure on staff during the pandemic, the contagious nature of the disease, and the considerable time pressure to implement a solution, these interviews were conducted remotely with only a minimum sample of available staff with various responsibilities to capture a wide range of requirements.

Since hospitals were dealing with a high demand for ICU beds [28], the aim of this study was to obtain important information to help us understand what design aspects of this tool could help to reduce the workload of clinical staff [4]. Such information could be used to design better tools to help with clinical decision-making processes [4]. Similarly, this may reveal a better understanding of which tasks performed by ICU staff may benefit the most from the introduction of such a dashboard. Further, we anticipate that understanding the various responsibilities of ICU staff could be a useful guide for future participatory design work focused on specific health care staff functions. Therefore, taking into account the preferences of end users for the dashboard design, including how data are displayed (eg, table, figure, graph), enables gaining a better understanding of the role for implementation of these preferences in a multifunctional and customizable dashboard, which could help to prepare ICUs for any future waves and mutations of the virus [29,30]. However, this may require further innovations in clinical processes. We are aware of the complexities of health information systems; therefore, the development and integration of new systems require careful consideration and human-centered design [23]. If this is not the case, there is a risk of developing new systems surplus to requirements, or causing further complications if staff need to switch between multiple systems. New systems could potentially compromise patient safety, be difficult to use and learn, and encounter resistance from staff, potentially resulting in poor uptake of the system [23,26].

Hence, the overall aim of this project was to collate a series of insights and requirements from end users across two ICUs to design and build a clinical dashboard to support their increased workload during the COVID-19 pandemic. Requirements elicitation, as seen in this work, by its nature is difficult, as requirements are volatile and necessitate translation from natural language via stakeholders through to tangible software [31,32], especially with a fairly unknown disease that continues to change, mutate, and impact society. The current pandemic has been an “extreme environment” [22] for researchers across disciplines. Hence, this work provides interesting insights regarding COVID-19 specifically but also provides an example of work conducted “in the wild” that encompasses the context, nuances, and uncertainties faced by both the researchers and the ICU staff [33]. The difficulties range from the time and additional pressure ICU staff were under at the time of writing but also entire workforces required to “work from home,” thus diminishing the inability to travel and collaboratively work together to previous expectations. Altogether, this provides a novel frame for this research to occur from initial planning, interviews, through to subsequent testing evaluation, and future iterations being deployed as this work is ongoing.

## Methods

### Research Questions

At a time of unprecedented workloads in the ICU, clinical staff time was in short supply. We interviewed six staff members working in ICU wards across two hospitals for the UK National Health Service (NHS) (Table 1). We were acutely aware of the additional strain on the NHS and staff; hence, we proceeded with interviews as a direct and simple method to capture requirements and reduce additional workload on ICU staff [31,32].

**Table 1:** Participant job role across the two intensive care units.

Participant ID number	Job title	Hospital
1099	Consultant in Intensive Care Medicine	A
1159	Sister (Band 7, Manager)	B
1252	Anesthetic Registrar	B
1587	Consultant in Intensive Care Medicine	B
1704	Consultant in Intensive Care Medicine	B
1839	Matron (Senior Nurse/Nurse Manager) in Intensive Care	A

Our exploratory interview questions were:

1. What tasks do each participant perform in the ICU, and where would a portable dashboard be helpful to improve safety or reduce workload on staff?
2. Do COVID-19 patients pose particular clinical challenges that need to be taken into account in the dashboard design?
3. Which data would staff need to look at on the dashboard and how should they be presented?
4. Would staff have any concerns about the introduction of a portable dashboard into the ICU?

### Participating Hospitals

The two hospitals participating in the study were distinct from one another, with one (hospital A) having a more technologically enhanced ICU where many systems are already digitized in comparison to hospital B, which remains more paper-based with a much larger ICU unit. The interviews were semistructured, lasting approximately 30-45 minutes, and due to the pandemic situation in early 2020 were conducted exclusively online. Topic guides were used to assist the interviewer and encourage consistency, with additional post hoc questions to further explore any potential themes. The interviews were recorded and transcribed verbatim. We used NVivo (version 12) [34] for the qualitative coding process and conducted an inductive thematic analysis [35].

### Analysis

To analyze these interviews fully, we took a multireasoning approach within our thematic analysis, which consisted of both deductive and inductive approaches to ensure rigorous development of coding trees [36,37]. Initially, we developed a preliminary codebook deductively via the data familiarization phase. We then entered a second phase that took an inductive approach to allow new themes to materialize as we coded each interview. We iterated through this process until the researchers reached a unanimous decision on the final codes, themes, and how they fit together. This in-depth and rigorous methodology aimed to ensure we were as thorough as possible for capturing requirements, alongside taking advantage of our multidisciplinary team, noting that the qualitative coders were not medically trained unlike other members of the team. Hence, it was important for us to ensure that we were accurate and appropriate in our codes, themes, and understanding [38,39]. Therefore, we sought medical insight and advice at every stage of the process within our team. We produced two distinct codebooks: one relating specifically to the requirements of a dashboard and another that related specifically to concerns of dashboard use in ICUs. The requirements codebook consisted of 96 codes that were initially subdivided into the following three requirement categories: 24 technical codes, 56 clinical codes, and 16 operational/logistical codes (see Multimedia Appendix 1). The concerns relating to the dashboard use codebook consisted of 24 codes, including 9 codes regarding design and 15 codes concerning operations (see Multimedia Appendix 2). The final codebooks were then used as the guide to code the qualitative interviews; therefore, in NVivo, each code would have words, phrases, and quotes from participants organized into these codes and themes. This ensured a straightforward and well-organized analysis. Hence, our hybrid approach [37,40] was the method employed for this codification, which identified existing patterns and subsequently regrouping codes into their emerging themes.

### Ethics Statement

This work was approved by the Faculty of Engineering Research Ethics Committee at University of Bristol (case 2020-3236).

## Results

### Overview of Key Requirements

Based on our interviews, we elicited five key requirements from a range of ICU staff to capture a wide range of roles and needs when using clinical dashboards. For instance, a dashboard must be adaptive and flexible to continue to be useful in changing clinical environments. Furthermore, dashboards need to be customizable because different staff may have specific parameters and information they need to see first (eg, “condition at a glance”). This can be achieved by ensuring there is some customizability for individuals or staff groups to select the information that is displayed. A dashboard would need to be mobile, which could reduce reliance on paper-based forms with patients. This also relates to task and patient management, where several staff noted that a dashboard could help with data entry and management alongside assisting with patient handovers, as all information will be collated into one system that is easily accessible. In contrast, some concerns were raised, for instance around infection control when carrying devices in and out of ICUs and high-risk-of-infection areas.

### Requirements for an ICU Dashboard

#### Flexibility With Changing Protocols for an Evolving Disease

Unsurprisingly, the new challenges posed by the COVID-19 pandemic featured heavily in the participants’ responses to our interviews (see Multimedia Appendices 1 and 2). As mentioned above, as these interviews were conducted in early 2020, relatively little was known about the nature of this disease and how to contain or treat it. This had direct implications for hospitals, highlighted by Interviewee 1587 (ICU Consultant), who described ICU staff difficulties handling COVID-19 patients while “keeping on top of constantly changing best practices.” At the time, no consensus had been reached regarding the clinical management protocols for COVID-19 patients, with staff under pressure to run additional tests while keeping track of the results. According to Interviewee 1704 (ICU Consultant), this absence of clear protocols created a very difficult working environment for staff:

…for a couple of days when I was on the COVID-19 side, full witness to it, it was a bit of a shambles and very stressful for the staff. There was no harm done to any of the patients, but it was asking staff to work outside their comfort zone and people found that professionally very difficult…

As ICUs are data-intensive environments by their nature, while in the process of adapting to a new disease and ever-changing protocols, a dashboard could help avoid staff forgetting to check certain parameters or simply avoid them due to being overwhelmed with new tasks and information. For instance, Interviewee 1099 (ICU Consultant) stated: “[a dashboard has the ability to] draw[s] my attention to things that I would probably forget about if I’m honest. I can only process X amount of information.”

One of the great challenges for medics and hospitals during the pandemic has been keeping on top of the new, fast-moving information and updates regarding COVID-19 management and treatment:

We are still learning a lot about [COVID-19 patients]. When [information] was coming from Italy, there was a lot of talk about how the patients were and how we were supposed to treat them, pretty much everything we were told has been wrong. It seems to be a very unusual disease and it’s not like anything we have seen. Whilst we were told it was a really bad pneumonia, which we had to treat with aggressive ventilation; it turns out it seems to be a disease of blood clotting, that affects the lungs. It makes our treatment that we are doing the wrong thing and potentially even harmful, so we’ve changed a lot about what we are doing…

Interviewee 1704, ICU Consultant

It’s a new disease process. We are learning all the time; best practice of evidence is constantly changing so keeping on top of that is difficult. […] Accessing best practice can be difficult, we have a new intranet […where] we have our single page checklist with guidance for these patients that is shared with [redacted]. I was thinking about what sort of things would be helpful and actually because it is a new disease process there is quite a lot of new things we don’t normally do, so a lot of regular blood tests that happen for example at days 1, days 3, 5, 7, keeping on top of when those are. They are all on our daily checklist, on our management guide. They are tests we wouldn’t normally do, but they are looking for specific things. Other new disease processes on top of the COVID, like HLH [hemophagocytic lymphohistiocytosis] that can happen, so sort of screening for those.

Interviewee 1587; ICU Consultant

Interviewee 1704 (ICU Consultant) further noted the continuous operational changes occurring in the COVID-19 “pods,” defined by Interviewee 1587 (ICU Consultant) as the “designated COVID-19 areas” where additional personal protective equipment (PPE) is required for entry, which created additional psychological and physical strain:

The COVID pod has changed quite a bit because advice on personal protection has changed. Initially, all the patients were in closed rooms with the doors shut and you had to put all the equipment on to go in and see them, […]. Now, everybody is wearing it all the time in the hall throughout the unit and the doors are open. So, when the doors were shut you didn’t do the ward round at the bedside, you were stood outside looking at all the data and the nurse inside was writing stuff on a white board to show somebody on the outside to write on the piece of paper. That wasn’t a sustainable solution because it was quite staff intensive.

[when health care staff move into the] high-risk areas […] there is a barrier psychologically to going into the room.

The accounts of participants illustrate the importance for an ICU dashboard to have the capacity to be flexible and easily updated due to constantly changing protocols, information, and advice (eg, reminders to check for specific parameters, changes in protocols regarding closed rooms or designated COVID-19 pods). This could draw from national- and hospital-level advice or information that is pushed out to staff via the dashboards pulling from various NHS information systems. This is a key requirement to ensure the dashboard continues to be usable as we learn more about COVID-19 and to potentially adapt this device into a tool that can remain integrated into ICUs more generally. These narratives highlight the ICU staff needs for an adaptable dashboard that can be updated with constantly changing real-time data about patient parameters, with new and revised routine alerts for new tests, and with reminders for specific trends to look out for when dealing with COVID-19 patients.

#### A Mobile Dashboard

Interviewees 1252 (Anesthetic Registrar), 1587 (ICU Consultant), 1704 (ICU Consultant), and 1839 (Matron) stated a preference for a dashboard that they would be able to use while walking around the ICU to attend to patients:

There is a lot of walking around by the nurse in charge, just to touch base with people for support and things. They are not static, so it would have to be a mobile solution

Interviewee 1704, ICU Consultant

A lot of my clinical duties are mobile so to not have the technology follow me and having to use fixed desktops is sometimes quite frustrating. As I say I am mobile, the whole of clinical care is conducted in a very mobile fashion

Interviewee 1252, Anesthetic Registrar

Thus, the dashboard would potentially replace paper forms and allow for simple and efficient data entry (both qualitative and quantitative) as staff move around while on shift. Interviewee 1704 (ICU Consultant) discussed new issues that arose specifically from dealing with COVID-19, such as the heavy reliance on paper forms in their hospital, given that computers are situated in high-risk areas, which, due to PPE use regulations, makes access to computers difficult. Hence, having a dashboard with remote access to various hospital systems and records that can also be used in mobile devices and taken by staff members outside of the pods would be helpful.

The other thing that COVID-19 has bought in with it, is our clerical staff […] who input a lot of data onto the computer, they’re not able to go into the area because they’re not fit tested with the masks. We are taking pictures of the observation chart with the iPads and uploading them to [redacted] [….] for them to then look at remotely, which is a bit of a fudge. It’s not brilliant, but it’s better than nothing at the moment.

By reducing paper forms and collating information neatly in one place, this would ensure information can be carried from patient to patient around the ward and would simplify a series of traditionally offline protocols, task, and data management. While simple in concept, realistically bringing together several diverse information systems and data across hospitals into a unified system or database is a highly complex task and may be difficult to integrate fully into the workforce [41]. However, examples of publicly available, deidentified EHRs do exist, such as the Medical Information Mart for Intensive Care (MIMIC) as part of the Beth Israel Deaconess Medical Center [42].

#### Customizability and Usability

A key theme arising from the interviews was the users’ needs to customize the dashboard (see Multimedia Appendix 1). For example, Interviewee 1099 (ICU Consultant) expressed a need for customization to support the different tasks and roles of the ICU staff, since clinical information such as patient parameters is crucial to performing the Intensive Care Consultants’ tasks, whereas operational data such as duration of patients in prone position would not necessarily be of interest to doctors yet would be of great importance for nurses. Similarly, Interviewee 1252 (Anesthetic Registrar) suggested the usefulness of knowing the patients’ pending and past procedures. Hence, the data required for a dashboard to be useful are extensive, complex, and would draw from several hospital information systems, including (where * denotes parameters that were also mentioned as important to view over time as trends and ^ denotes markers particularly important and of interest for COVID-19 patients): C-reactive protein (inflammation marker)*^, D-dimers (blood clotting marker)*, ferritin (inflammation marker)*^, lymphocyte count (inflammation)*^, platelet count (inflammation)*^, procalcitonin levels (inflammation)*^, pending tests for specific HLH patients, blood pressure*, white blood cell count*^, fraction of inspired oxygen, oxygen level*^, oxygen supply level for personalized care, peak airway pressure for COVID-19, tidal volume size, positive end-expiratory pressure^, arterial oxygen/inspired oxygen ratio (PF ratio), plateau pressure^, type of ventilation, Sequential Organ Failure Assessment (SOFA) score, Glasgow Coma Scale score, intracranial pressure*, ventricular tachycardia^, infusion rate of vasopressors*, number and absorption of nutrition calories, and COVID-19 status. Further, specific requests for alerts to be associated with specific data were noted, such as abnormal values across parameters, 7-10 days of static oxygen (meaning a computed tomography pulmonary angiogram scan can be completed), pending tests for COVID-19 patients, clinical deterioration, and pending procedures for patients. Finally, other information was requested that was more operational in nature, including bed layouts, number of patients on dialysis, nurse locations, patient numbers across units, patient flow information (eg, admission, discharge, changing of units), and relevant patient handover information.

Unsurprisingly, a lot of data and information were requested to appear in the dashboard, which may be overwhelming and difficult to navigate or simply not of relevance or interest to certain roles in the ICU. Interviewee 1099 (ICU Consultant) suggested that a fully individualized dashboard for each staff member would be ideal as this allows for a tailored configuration for each of the roles within the ICU. However, it is important to be cautious when implementing highly customizable systems, since this customizability may induce errors when overstressed staff are required to make fast decisions. Hence, careful consideration is needed to determine what degree of customization is advisable for this device at the user level. It is important to have functionality that allows additional staff access on an ad hoc basis. As Interviewee 1587 (ICU Consultant) stated, staff from different areas of the hospital may need access to the dashboard. For example, COVID-19 patients often need nutritional and dietary assistance; hence, nutritionists may also need specific systems access for these patients.

Alternatively, Interviewee 1839 (Matron) suggested having a split view where parameters could be presented as broader categories such as “Clinical Parameters” (eg, ventilation, tidal volumes, dialysis) or “Safety Parameters” (eg, delirium, infection, prone position turns), so that these measures could be useful for various health care staff functions and responsibilities. However, this could also be problematic if functionality allows for toggling role-specific parameters and the lack of prima facie data may cause staff to miss trends in parameters not shown on the screen.

Several participants noted the importance of integrated systems (see Multimedia Appendix 1). For example, Interviewee 1099 (ICU Consultant) stated their current systems necessitates that they “have to open up 5 different screens to get the data [they] need and that is pretty labor-intensive,” or that they are “often chopping and changing through different programs…,” which demonstrates the importance of having an intuitive interface that consolidates relevant data on demand. Similarly, in terms of usability, it is problematic if a system requires additional “administration” for staff to find information and results on pending tests. This can be illustrated with the following account from Interviewee 1099 (ICU Consultant) about the [redacted] system:

When you have somebody who comes in with a chest infection, 15 tests are ordered: five aren’t back, five were never sent, and five are back, but you would never know that by looking at [a system]. You would only know that by looking at this separate program.

It is therefore crucial for the dashboard to be well-integrated with other hospital systems to avoid data and work duplication. It is thus paramount to ensure that a new dashboard does not add complexity but reduces workload to access data by extracting it from the existing systems. It is essential for staff to be able to customize their view to quickly sift through large amounts of information, understand patient needs, and determine next steps. As a requirement, this is achievable and realistic, as it is common for information systems to have user profiles with individual logins [43]. Further, having default user profiles based on roles and grades within the workforce is a reasonable requirement to implement, where individuals can request additional accesses ad hoc. However, the level of customizability offered alongside user profiles would require additional testing and research (eg, changing color schemes, data access changes).

#### Dashboard Layout and Trends of Incoming Data

##### Patient Overview

In general, there was a tendency for all participants to comment on how data should be processed and presented on the dashboard. Interviewee 1839 (Matron) stated a preference for the ability to view the whole ward (which could also track bed and patient expansion). If this were to match the physical layout of the beds, this would be useful to find patients quickly and to effectively plan a patient’s acuity (level of nursing care) quickly. This is especially important should the unit become busy or indeed require a quick and large-scale expansion in patient numbers, as envisaged at the UK’s “Nightingale” hospitals. In addition to the ward view, Interviewees 1099 (ICU Consultant), 1704 (ICU Consultant), 1839 (Matron), and 1252 (Anesthetic Registrar) suggested the inclusion of a summarized “Condition at a Glance” view, which would allow for those starting their shift to quickly get up to date. This was echoed by Interviewees 1704 (ICU Consultant) and 1839 (Matron), who suggested a display of “overarching” parameters of all patients on the ward.

Interviewee 1587 (ICU Consultant) reported a similar requirement that would display the most critical parameters (eg, SOFA scores, tidal volume), which would allow the Intensive Care Consultants to see the trend of a patient’s current condition. Interviewee 1587 (ICU Consultant) argued that calculating SOFA scores is an arduous task for junior doctors; hence, making these calculations available and easy to interpret in a dashboard will save ICU staff time and cognitive energy [44].

Of course, there is more to ICU patients’ health care than a first-glance interpretation of these parameters. Interviewee 1839 (Matron) stated the complexity of patient monitoring when patients might appear fine in terms of typical baseline metrics, but in reality, their actual state is misrepresented by data: “[a] patient could be fine, but they’re on a lot of inotropes (eg, noradrenaline) or ventilation, and it looks OK, but they are on 100% oxygen and/or quadruple noradrenaline.” The intuitive assessment required in such cases could only be achieved through an appropriate identification of the daily and hourly trends by the ICU staff, of the patients’ inflammatory markers and oxygen levels, according to Interviewees 1704 (ICU Consultant) and 1587 (ICU Consultant). Thus, the availability of these data on a dashboard will be particularly important for health care staff to decide on COVID-19 patients’ clinical care and to devise provisions for their safety, as expressed by Interviewees 1099 (ICU Consultant) and 1704 (ICU Consultant). Further, Interviewee 1587 (ICU Consultant) discussed the significance of data trends, especially regarding COVID-19 patients, such as decisions about when patients can be weaned off ventilation as well as monitoring the prone position ventilation timings. Additionally, a dashboard could track specific sets of data, which may offer statistical insights (Interviewee 1252, Anesthetic Registrar) into how to better treat future patients for particular diseases or conditions, as these data will be able to provide baselines and expectations, particularly for new diseases such as COVID-19.

##### Data Visualization, Warnings, and Alerts

There were differences among ICU staff as to how they wanted data to be presented in this type of tool (see Multimedia Appendix 1). A suitable example of this would be the informants’ preferences for graphical displays that would help to address two important issues pointed out by Interviewee 1587 (ICU Consultant):

I think if you click on it, you can see a graph, but to be honest we don’t regularly do that. Ninety percent of the time it’s just numbers completed on a sheet…We literally have a piece of paper that junior doctors fill in in the morning or if they haven’t, we go on [redacted] and click on the CRP trend on there and see what’s happening. You can get a graph of it, but it’s not ideal.

Hence, it is important to enable users to have a degree of autonomy to customize the dashboard for individual patients (Interviewee 1099, ICU Consultant), and to transform data and information into a format that best suits their learning needs and information processing style. Alongside ways to visualize data, it is critical to have suitable real-time deterioration alerts both for clinical and safety parameters, which may include visibly highlighted alerts on abnormal values, as well as real-time alerts on staff deviations from practice (Interviewees 1099 [ICU Consultant] and 1704 [ICU Consultant]). However, when it comes to the display of these warnings, Interviewee 1099 (ICU Consultant) discussed what would be the most suitable parameters for COVID-19 patients (eg, PF ratio and driving pressure) to help clinicians decide when to start weaning the ventilators, as well as the adequate time parameters for the graphic view. This interviewee stated the importance of including suitable “cutoffs” in terms of data presentation, because to plan COVID-19 patient care, Intensive Care Consultants must consider both the presence of abnormal values as well as how these values behave over time (trends). In addition, Interviewee 1839 (Matron) noted determining thresholds for colors and notifications is not a trivial matter, especially if they need to be tailored to specific medical conditions or personalized to each individual patient. Therefore, a dashboard that presents data in a way so that the user could see a longer patient history may be extremely helpful. This could be facilitated by allowing the graphs to be scrolled through horizontally to show earlier data and see longer longitudinal trends since the patient’s admission.

It is important to be aware that notifications or alarms are extremely common in ICUs due to the variety of abnormal values of health parameters in critically ill patients. Therefore, when designing a new dashboard, a reasonable balance is needed to avoid “alarm fatigue” and to prevent staff from missing patient deterioration markers, which can lead to detrimental outcomes [45-48].

The dashboard would give me the triggers to go sniffing around into the detail of the data […], I would just highlight the noradrenaline box and the base excess [and hide other parameters and] look at those two things. What’s the trend? […] I think the personalization of being able to manipulate it on one screen…

Interviewee 1099; ICU Consultant

trends are brilliant. […] it doesn’t really matter what the noradrenaline is, if it has doubled in the last hour, it’s not a good thing. [...] Actually, it’s the step change that is the important thing. For me, I was straight drawn into the color change and the arrows, then just lost sight of the numbers a bit. That is probably a good thing because I would then go looking into that patient detail on the system to see why their noradrenaline is going up and doubled. So that was quite a good trigger.

Interviewee 1099; ICU Consultant

Here, the requirement relates to the layout of the dashboard and how data are presented (with some in real time). This called for flexibility in terms of how data can be graphically displayed to suit the staff member using the device, which may also help with the number of alarms and notifications in the ICU. This requirement is relatively simple to implement within a dashboard system, where users will be able to shift between graphic and tabular displays of information or seeing longer-term trends of a patient, for example. However, attempting to visualize data and highlight when parameters are shifting negatively for the patient is inherently more complex; thus, testing and examining what the thresholds should be are crucial to reduce alarm fatigue among additional stress for staff. This would include investigations regarding individualistic measures versus overall baseline “cutoffs.”

#### Task and Staff Management

##### Patient Handovers

A key theme regarding staff and task management were patient handovers (see Multimedia Appendix 1). Handovers (both regarding staff shifts and turnover of their allocated patients), are complex two-way processes between a variety of staff signing out and updating those coming in to take over, where the accuracy and effectiveness of this information exchange “will facilitate consistency and continuity of care” [49]. This is particularly important for critical care patients, where omission of pivotal information during the handovers could influence future treatment and subsequently cause failures in patient management [49]. Since the use of a dashboard could rapidly help to capture and track wider information regarding patient status and care requirements, it is evident that the implementation of this tool in the ICU environment could facilitate more structured and effective patient handovers. For example, Interviewee 1839 (Matron) stated:

We know that handover time and transfer of care is a pinch point where if there is going to be an issue or problem occurring, we often track it back to that point in time. Where something has been missed, not handed over or at that point they may look at something and go “that’s not what I remember it being.” That’s the trigger to go back systematically through all their different bits. Or a doctor has come along and changed the rate of a pump and not told somebody. We know that is a really pivotal time so some kind of overarching view of the main clinical elements of a patient care would be helpful. That would give them a visual aid to that and anything that would help a hand over of care, would absolutely be welcome.

Similarly, Interviewees 1099 (ICU Consultant) and 1839 (Matron) coincided in stating the importance of allowing time for scrutiny of new inputs of their colleagues and data updates from various patients that have been handed over. However, as Interviewee 1099 (ICU Consultant) pointed out, outlining changes in patient parameters during quick handovers is done with great difficulty while having to navigate multiple hospital systems to gather the information required:

I had a handover from my colleague - but I want to process it in my own mind and want to see what’s changed over the last 12 hours, since they handed over, [currently] I have to open up 5 different screens to get the data I need and that is pretty labor intensive.

##### Data Entry

Due to the important consequences at stake, extreme care and monitoring are exercised in the ICU environment to ensure accurate data input in their systems.

If you are feeling responsible for the patient, which you are as a consultant, you need to double check that [data, patient notes]. The only way to do that is to physically look at those [systems] and paper notes yourself

Interviewee 1099, ICU Consultant

Moreover, when planning patient care targets, ICU staff in managerial positions must carefully balance patient-management workloads of staff with their data input tasks. This links in with comments from Interviewee 1099 (ICU Consultant) when expressing concern about overtired staff with data entry responsibilities such as recording general observations about patient progress and invasive procedures, among others:

[staff] will be absolutely knackered at three in the morning and just put [in] the bare minimum. They’ll go, “patient had an operation, and this is what happened” and forget about other stuff.

On these grounds, participants considered having a tool that can assist staff in the transcription and modification of patient data with a minimum error rate to be important. Arguably, errors may continue to occur in the presence of a digital interactive dashboard; however, research has shown that using digital systems to collect and log data (rather than pen and paper) reduces errors in data recording and data entry [50,51]. The use of a dashboard helps to provide a faster way to populate handover or debrief notes. Furthermore, Interviewee 1252 (Anesthetic Registrar) noted the lack of any formal system at present to document any medical advice provided to patients over the phone. This could be a simple note about the patient that can be added to the dashboard to ensure an overview of all advice and information previously provided to the patient.

##### Task Management

A major issue reported by the participants was a lack of warnings in their current systems about forthcoming completion times of pending tasks and targets, which could be built into a dashboard. Interviewee 1099 (ICU Consultant) stated that a careful balance must be struck with off-target warnings to avoid undermining staff confidence:

The warnings that are built into the target need to be in advance, there is no point telling people at midnight you’ve not met your fluid balance target because it will just demoralize people.

Consequently, it is unsurprising that one of the participants’ most frequently mentioned dashboard requirements was to have warning notifications ahead of completion times (see Multimedia Appendices 1 and 2), which would certainly work as a task management system that will ensure timely completion of the multiple pending tasks and daily targets of medical and nursing ICU staff, including ventilation weaning, daily prone and supine ventilation sessions for COVID-19 patients, and other safety tasks of nursing staff; invasive procedures such as tracheostomies; monitoring pending microbiology and specific tests for COVID-19 patients; changing of drugs; and speaking to relatives (Interviewees 1099 [ICU Consultant], 1159 [Sister], 1252 [Anesthetic Registrar], 1587 [ICU Consultant], 1704 [ICU Consultant]), among many other responsibilities. Interviewee 1099 (ICU Consultant) stated that this type of task management system would give managerial staff peace of mind by knowing that “loops are closed,” especially when the ICU becomes extremely busy and “people forget about minutiae.” Furthermore, in a context of ever-changing guidance and information, Interviewees 1159 (Sister) and 1587 (ICU Consultant) also agreed that it would be extremely useful to have daily task checklists (eg, safety checks) as a suitable requirement of the dashboard, which should also include enabling inputs of data as needed. Interviewee 1587 (ICU Consultant) also pointed out how having a dashboard to prompt staff members to finish tasks would be important to address the absence of warnings on pending targets in their current system, especially with new diseases such as COVID-19 when more tests than usual are frequently needed. For example, Interviewee 1099’s account illustrates how ICU staff struggle to juggle their immediate tasks with their daily patient care targets for both COVID-19 and non-COVID-19 patients:

There will be a patient that will be on multiorgan support, have to go for a scan, have to go to theatre, come back. Then the nurses will try to make sure the patients are fed, […], all sorts of complex care issues [are] going on. That [eg, fluid] target then drifts into the background. There’s no prompt to say, “your fluid balance is nowhere near target and you have four hours to go. What are we going to do to solve this problem?”

To obtain a general picture of the ICU patient care flow, interviewees discussed the advantages of having an overall view of staff numbers and their corresponding workload, alongside data about patient admissions and patient flow (eg, patient discharge and transfers either for tests or to other wards), as pointed out by Interviewees 1159 (Sister), 1839 (Matron), 1704 (ICU Consultant), and 1252 (Anesthetic Registrar). For example, the following testimony of Interviewee 1159 clearly portrays the managerial ICU staff need of a well-updated and integrated system showing workload allocation:

We need a way of knowing which nurse is in each bed space so if there is an issue, we can speak to that nurse looking after that patient. If it was two shifts down the line, there was something we needed to get hold of somebody about.

At the same time, from the following account of Interviewee 1704 (ICU Consultant), it can be inferred that managerial staff are simultaneously responsible for overseeing the staffing of the unit while monitoring the changing conditions of all patients. Hence, the requirement for a new system to collect these two categories of data was mentioned:

They collect this data on a piece of paper as well and it’s in pencil so they can rub stuff out and change it. I have been trying for years to get iPads for them to use, we need some kind of software for that. That data would be really important to analyze: the patterns of activities during the day, to optimize our staffing models or things like that.

This statement highlights that the implementation of a dashboard for ICU use could provide a much-needed opportunity to shift from pen and paper to a digital system of data collection and monitoring, alongside a new strand of data analysis that could help optimize staff time and workload. Interviewee 1252 (Anesthetic Registrar) provided additional corroboration of a dashboard’s value to mobile staff such as Registrars by having an effective oversight of all patients pending transfers to other wards and for tests (eg, for a computed tomography scan or other tests). This relates to Interviewee 1704’s (ICU Consultant) statement about the need to better optimize staffing models and the daily distribution of tasks by accurately monitoring staff workload and whereabouts as follows: tracking staff timings for patient care for each allocated patient, as well as producing continuous insight into the location of the health care staff throughout the different ICU wards during their shifts (Interviewees 1159 [Sister] and 1252 [Anesthetic Registrar]). According to Interviewee 1252 (Anesthetic Registrar), this two-fold patient-staff tracking system would be very useful for staff who are constantly busy (Registrars and Running Nurses). With this new system, they can efficiently share their work in relation to their location, the patient transfer destinations, and the numbers of daily transfers.

This final requirement, which touches on all prior requirements for the dashboard, is ensuring that the dashboard is seamlessly integrated with other hospital systems. In this way, staff can access additional, external data (eg, authorized views of blood test results from other hospital systems). This would help to tackle the issue described by Interviewee 1252 (Anesthetic Registrar) when characterizing the process of patient admissions from other hospitals as “data heavy” with a lot of “transcribing of various different sources onto the intensive care unit systems,” where data are currently not being pulled neatly into one system. As stated previously, this is a highly complex task that would be difficult to integrate as current systems are siloed across hospitals [42].

### Concerns About Dashboard Use

As shown in Multimedia Appendix 2, there were two main operational concerns among ICU staff regarding the use of dashboards. The first concern, raised by Interviewee 1587 (ICU Consultant), related to the potential increase of infection via use of mobile technology and equipment. On the assumption that these dashboards will be used in mobile devices such as iPads, Interviewee 1587 (ICU Consultant) questioned whether these devices should be allowed into the infection-controlled areas of the ICU, specifically at the bedside of COVID-19 patients. As possible solutions, Interviewee 1587 (ICU Consultant) mentioned the use of disinfecting wipes for mobile devices, but mostly adopting clinical protocols that avoid the need for using a device at the bedside.

We don’t take the iPads in to see patients. In terms of risks, they are potential fomites, a sort of vector for transmission of infection. We wouldn’t take them into bed spaces. Much like cleaning our phones, we are good at cleaning with special Clinell wipes. Yes, there is a potential risk… We stand outside where it is lower risk. We cluster round as a ward round to write up our notes and decide the plan. Only one of us will go in to examine the patient. Everyone else will wait outside.

The second operational concern regarding dashboard use related to the differing levels of technology literacy among ICU staff. For example, Interviewee 1099 (ICU Consultant) stated that unless there already is a culture around the use of dashboards and technology, encouraging staff to actually use and engage with this type of tool might be difficult [27]. This is an interesting comment as Interviewee 1099 is based at the more technology-enhanced ICU that uses dashboards among other devices regularly, where such tools are integrated. Hence, these concerns additionally relate to the managing expectations in staff (Interviewee 1587, ICU Consultant) of what the dashboard will do, how it should be used, and protocols regarding these devices, as it is important not to oversell a new technology’s potential impact for the ICU.

Finally, there was some ambivalence from staff members about the dashboard design, as illustrated by the concerns raised by Interviewee 1099 (ICU Consultant), who is based at an already technology-enhanced ICU, in relation to what should be the “acceptable” parameters for the notification and warning timings with regard to both COVID-19 patients and staff targets. Interviewee 1099 (ICU Consultant) also raised concerns regarding notifications or alerts coming in at inappropriate times, since this could cause the adverse effect of “demoralizing people.” This participant further mentioned a concern for having a tool with which staff could compare their unit target achievements with other units. This could increase ICU staff stress, and might lead to suffering from burnout or cognitive overload, “wow, mine’s [targets or parameters] all red, things aren’t going very well,” which could have ramifications for both patients and staff due to the well-documented fact that the ICUs are incredibly stressful environments [5,6,52]. These are important concerns to address early and to ensure staff are all fully informed regarding the system itself and the transparency regarding how the digital logs that it will produce may be used outside of patient monitoring (eg, can these be used to assess staff performance in the workplace?). Hence, when implementing new systems, the engagement of end users is key to ensure expectations are set and staff can feel supported by these new systems.

## Discussion

### Principal Findings and Conclusions

In response to the critical situation of two local ICUs, we conducted a series of interviews to elicit requirements for a bespoke dashboard to help ICU staff save time and work more efficiently, particularly during the COVID-19 pandemic. We found that despite having limited access to end users, our approach of conducting remote requirements interviews for developing a dashboard for COVID-19 ICUs has been successful. The rapid cycle of interviewing end users, prototyping the user interface, and iterating over the software design, despite taking place in extreme and distressing circumstances of the pandemic, has proven to be an effective way of producing functional software requirements. These requirements have in turn allowed for the development and deployment of an interactive dashboard currently being tested and evaluated across two hospitals.

The first requirement was the need for a flexible dashboard, primarily to help ICU staff respond to rapidly changing guidance for the management of this new disease. The second requirement emphasized the need for a mobile dashboard, which allows staff to walk around wards with real-time data and information of patients. The third requirement focused on customizability of a dashboard, stemming from the great diversity of roles and tasks conducted by ICU staff. Related to this was the fourth requirement, which was the ability to track and visualize real-time data and daily/hourly trends on patient parameters. The fifth requirement was aimed at pending tasks and targets for staff management. All requirements highlight a need for the integration of different hospital systems within the dashboard, which is a longstanding challenge in medicine [41]. Alongside these requirements, participants raised concerns regarding the infection-risk safety issue of bringing devices into the ICU and of the timing of warnings and alerts.

The study findings confirmed that digital solutions for ICU use would potentially reduce the cognitive load of ICU staff and reduce clinical errors at a time of notably high demand of intensive, critical health care [17]. As summarized by Interviewee 1099 (ICU Consultant), the beneficial implications of having this dashboard would hopefully be that “not only will it make the system more efficient” but it will further give them the possibility of “looking after more patients more safely.”

### Limitations

We acknowledge that the sample size is small due to the workload on ICU staff caused by the COVID-19 pandemic, which was the underlying motivation for this research. However, we did capture requirements, perspectives, and experiences from a wide range of clinical roles within the ICU environment across two somewhat different hospitals, particularly from those heavily involved in the health care of COVID-19 patients during the pandemic. There are a number of ways we could have elicited the requirements for the dashboard, including from questionnaires, joint application development, storyboarding, and protocol analysis [31,32]. However, with the time pressure to develop a dashboard that was working and usable, alongside the time pressures ICU staff were under, we wanted to continue with the most straightforward and least cognitively heavy method of elicitation for the ICU staff.

Further, we are aware that our dashboard will require extensive testing in the ICUs with end users such as our interviewees to refine the design and functionality. This would include examining how we would tailor the dashboard for different roles (eg, having a home page with various types of information for each role such as nursing staff vs a consultant or a nutritionist). This will be an iterative process, where we acknowledge that not all needs will necessarily be met; however, our aim is to ensure the device is usable and enhances staff.

We are also aware that our sample comes exclusively from the region of Bristol, which may not be representative for ICUs across the rest of the United Kingdom or indeed outside of the United Kingdom. However COVID-19 has impacted health care provision in many regions and many countries worldwide, and many of the staff, patient, and task management requirements; the ability to track and monitor trends; and the dashboard customization for individual staff members are likely to be common requirements both across the United Kingdom and around the world [53].

## Appendix

Multimedia Appendix 1 Code tree classification of dashboard information preferences (96 codes).

Multimedia Appendix 2 Code tree classification of concerns about dashboard use (24 codes).

## Abbreviations

EHR: electronic health record
HLH: hemophagocytic lymphohistiocytosis
ICU: intensive care unit
MIMIC: Medical Information Mart for Intensive Care
NHS: National Health Service
PF: arterial oxygen to inspired oxygen ratio.
PPE: personal protective equipment
SOFA: Sequential Organ Failure Assessment
